# The impact of digital technology on psychological treatments and their dissemination

**DOI:** 10.1016/j.brat.2016.08.012

**Published:** 2017-01

**Authors:** Christopher G. Fairburn, Vikram Patel

**Affiliations:** a Oxford University, Department of Psychiatry, Warneford Hospital, Oxford OX3 7JX, UK; b London School of Hygiene and Tropical Medicine, UK

**Keywords:** Digital health, Digital technology, Psychological treatment, Internet, Blended treatment, Training, Dissemination

## Abstract

The psychological treatment of mental health problems is beginning to undergo a sea-change driven by the widespread availability of digital technology. In this paper we provide an overview of the developments to date and those in the pipeline. We describe the various uses of digital interventions and consider their likely impact on clinical practice, clinical services and the global dissemination of psychological treatments. We note the importance of online clinics, blended treatment, digital assessment and digital training.

## Highlights

• Psychological treatments are undergoing a sea-change driven by digital technology.
• New forms of treatment are being developed.
• Existing treatments are being converted into digital interventions.
• Blended treatment is gaining in popularity and it can take a variety of forms.
• Autonomous digital treatments have potential as public health interventions.

## 1 Introduction

The psychological treatment of mental health problems is beginning to undergo a fundamental change. This change is being driven by the widespread availability of “digital technology” by which we mean computers, the internet, mobile devices such as smartphones, and mobile software applications (apps). In this paper we describe the various uses of digital interventions and consider their likely impact on clinical practice, clinical services and the global dissemination of psychological treatments.^1^

## 2 Digital treatment

There are well established digital treatments for depression and most of the anxiety disorders, and for problems such as insomnia (Andersson & Titov, 2014). The great majority are self-help programmes designed either to be used on their own or with some form of support. These treatments vary markedly in their content, clinical range, format, functionality and mode of delivery.

### 2.1 Content

The majority of the digital treatments are forms of cognitive behaviour therapy (Andersson, 2014). Most have been derived from existing face-to-face treatments or from self-help books based upon them. Some are greatly simplified versions of the original treatment and are little more than collections of “tools”, whereas others retain both the treatment's procedures and the strategies that govern their use. In general, the interventions make more use of behavioural than cognitive procedures and often there is a prominent educational component. Indeed, some interventions present themselves as educational programmes rather than treatments, and deliver the intervention in “lessons”, not “sessions”.

There are digital versions of other forms of psychotherapy including acceptance and commitment therapy (Pots et al., 2016), behavioural activation (Ly et al., 2014), interpersonal psychotherapy (Donker, et al., 2013a), mindfulness interventions (Spijkerman, Pots, & Bohlmeijer, 2016) and problem-solving therapy (Kleiboer et al., 2015). These adaptations have received less research attention than the cognitive behavioural ones. Like the cognitive behavioural interventions, they vary in the extent to which they retain the strategies and procedures of the original treatment.

Truly novel digital treatments are few and far between. Examples include positive cognitive bias modification as a potential treatment for depression (Blackwell et al., 2015), virtual reality-based exposure in the treatment of anxiety disorders (Valmaggia, Latif, Kempton, & Rus-Calafell, 2016) and persecutory delusions (Freeman et al., 2016), and the use of robotic technology to improve social interaction in autism spectrum disorders and dementia (Riek, 2016). An example of an intervention that is still at the experimental stage is the use of a computer game to block the reconsolidation of intrusive traumatic memories (James et al., 2015).

### 2.2 Clinical range

The existing digital treatments differ in the breadth of psychopathology that they address. Most are disorder-specific but a few are even more precisely targeted such as one designed for people with suicidal thoughts (Van Spijker, Van Straten, & Kerkhof, 2014). Conversely, some are “transdiagnostic” in scope and have a broad clinical range (Craske et al., 2009, Titov et al., 2011). These have great potential clinical utility.

### 2.3 Format

The interventions vary in their format. Some retain that of the face-to-face treatment from which they were derived; for example, by having regular weekly sessions. (In digital treatments “sessions” are times set aside by the “user” (“patient”, “client”) to devote to the intervention.) Others modify the format to match the way that websites or apps are typically used (Ben-Zeev et al., 2015). Generally, this results in briefer, more frequent, sessions than in face-to-face treatment, and often the overall length of treatment is shorter.

The interventions also vary in their structure. Some are linear, progressively leading users through the intervention step-by-step, whereas others have a variety of modules which may be used with partial or total flexibility.

There may be a degree of personalisation (“tailoring”). In practice, this may amount to little more than matching the text and clinical examples to the user's demographic group (e.g., middle-aged, female) although some interventions go further by incorporating algorithms that generate a treatment which matches aspects of the user's psychopathology. These algorithms are either derived from the strategy inherent to the treatment or the recommendations of experienced clinicians. In the future machine learning may be used for this purpose (Abdullah et al., 2016).

An alternative to tailoring is allowing users to select components of the intervention that suit their particular concerns. For example, people with depression might choose modules on Worrying, Difficulty Sleeping and Improving Concentration, as well as ones on Low Mood and Negative Thinking. Whether this “pick and mix” approach leads to a better outcome than a clinician-led or algorithm-led approach needs to be determined. The answer may depend upon the type of psychopathology being addressed.

### 2.4 Functionality

The early digital interventions consisted of little more than text and thus resembled printed self-help programmes. This is changing. More attention is being given to their appearance, appeal and ease of navigation, and the interventions may include features such as learning exercises, self-monitoring tools, progress reports, downloadable documents, audio- and video-files, audio- and video-feedback, avatars, quizzes and games. Examples of multifaceted and technologically sophisticated interventions are recent ones for social anxiety disorder (Stott et al., 2013) and post-traumatic disorder (Wild et al., 2016). A few interventions have been delivered entirely in the form of a game (Merry et al., 2012), a modality that may especially suit younger users and is attracting considerable interest.

Two innovations are likely to gain ground in the near future. The first is the use of virtual reality-based procedures as the equipment required is becoming more readily available, and the second is artificial intelligence-informed communication with the user.

### 2.5 Mode of delivery

A major way in which the interventions differ is in their mode of delivery. Most are delivered via websites and can be accessed on a wide range of devices, although some are not suitable for viewing on the small screen of the typical smartphone. Others are app-based and have been designed with smartphones in mind (Donker, et al., 2013b). Whether interventions are website-based or app-based has an influence on the form that they can take and the functions that can be used. Certain interventions employ both modes of delivery; for example, a website-based intervention may have an accompanying app for purposes such as self-monitoring or planning ahead.

## 3 Digital assessment

Digital technology provides new means of assessing and tracking psychopathology. At the simplest level, it can improve both the administration and interpretation of assessment questionnaires which, until recently, have been largely in pencil-and-paper format and manually scored. Many questionnaires are now available in digital form and can be completed via a website or app. This allows them to be automatically scored and interpreted with reference to established norms, and in some instances the scores can be transmitted directly to the clinician, the user and the user's clinical record.

The psychometric performance of digital questionnaires is a matter of concern as their presentation often differs from that of the source instrument. Overall, their performance appears to be similar to that of their pencil-and-paper counterparts but exceptions have been found (Alfonsson et al., 2014, van Ballegooijen et al., 2016). Little is known about the performance of app-based questionnaires. Some degree of standardisation of digital questionnaires seems desirable, perhaps by instrument developers creating “approved” digital versions, ones which have a consistent presentation across different devices.

Digital technology opens up new modes of assessment. Virtual reality procedures can assess sensitivity to particular environments (Freeman, 2008) and the presence of sensors in smartphones makes it possible to track many phenomena on an ongoing basis including sleep, movement, physical activity, speech, device usage and the person's location (Abdullah et al., 2016, Saeb et al., 2015). How to use this information is only just beginning to be explored. It may prove possible to catch new episodes at a very early stage and supply interventions at the very time that users might benefit most (Faurholt-Jepsen et al., 2014). “Real time” intervention might be especially relevant to suicide prevention if markers of imminent risk could be identified (Christensen, Cuijpers, & Reynolds, 2016). However, psychopathology tracking is not necessarily benign. It can magnify rumination and self-focus, and it has been reported to trigger emotional instability (Murnane et al., 2016). Similarly, the immediate delivery of interventions might create reliance upon them which could interfere with the acquisition of self-management skills.

## 4 Digital training

Digital technology is increasingly being used for educational purposes. Some “massive online open courses” (MOOCs) attract huge numbers of students from around the world (Eichhorn & Matkin, 2016). If it were possible to train therapists in a similar way, this would be an important advance as the conventional form of training is not scalable and is therefore a barrier to treatment dissemination (Fairburn & Cooper, 2011).

Clinician training websites are starting to be developed and evaluated (McHugh & Barlow, 2012). Some are basic in form and constitute little more than brief lectures or segments of text combined with video clips of therapy. Others describe the treatment in great detail and illustrate it with extensive libraries of video clips. The more sophisticated training websites also incorporate learning exercises and formative tests to help trainees grasp key concepts and master challenging procedures. Digital training has many advantages. In addition to letting trainees see therapy actually being delivered, it can be accessed whenever and wherever it suits the trainee, and it can easily be updated. Digital training programmes can be used on their own or supplemented with support.

Therapist training has been neglected as a research area (Fairburn and Cooper, 2011, McHugh and Barlow, 2012). Digital training is changing this. Training research can now be conducted on a much larger scale than before as digital training allows large numbers of geographically dispersed clinicians to be trained simultaneously. For example, a training website for enhanced cognitive behaviour therapy for eating disorders has been tested in a proof-of-concept study across an entire country; a RCT across North America; and in a global cohort study involving over 750 clinicians.

## 5 The research on digital treatment

Digital treatment has been the focus of an impressive amount of research given that this is a young field. Many of the website-based interventions have been tested in randomised controlled trials (RCTs) and their findings have been the focus of numerous systematic reviews (Andersson et al., 2014, Richards and Richardson, 2012). Surprisingly, app-based interventions have barely been studied (Donker, et al., 2013b). Here is a brief summary of the main findings.

1 *Direct-to-user digital treatments are popular and can access underserved groups*. A leading example is MoodGYM, a free online intervention for depression that has been available since 2001 (Christensen, Griffiths, & Korten, 2002) and has been used by over three-quarters of a million people. An important shortcoming of direct-to-user interventions is that completion rates are low if there is no accompanying support. Certain forms of psychopathology may prove to be more amenable to direct-to-user treatment than others. The eating disorders bulimia nervosa and binge eating disorder might be particularly suitable as binge eating is a repeated highly aversive experience which responds well to self-help interventions (Wilson & Zandberg, 2012) yet many sufferers do not seek treatment because of the associated shame and secrecy (Hart, Granillo, Jorm, & Paxton, 2011).
2 *Online clinics can obtain clinically relevant change on a large scale*. A good example is MindSpot, an Australian online clinic. In its first year of operation it provided supported digital treatment to over 2000 adults with depression or certain anxiety disorders (Titov et al., 2015). Over 70% completed the treatment with limited external input and their intent-to-treat outcome was similar to that of equivalent face-to-face treatments.
3 *Supported digital interventions are more effective than unsupported ones*. This is a consistent finding although, depending on the context and the specific intervention, the difference is not necessarily great (Baumeister, Reichler, Munzinger, & Lin, 2014). It is generally thought that the explanation lies in better treatment adherence in the presence of support (Mohr, Cuijpers, & Lehman, 2011).
4 *When accompanied by support, digital interventions are as effective as face-to-face treatments*. This is the conclusion drawn by several systematic reviews and meta-analyses (e.g., Andersson et al., 2014, Cuijpers et al., 2010). It is a important one as it has major implications for policy makers. As the conclusion is largely based on small-scale studies, most of which advertised for participants, it would be greatly strengthened by appropriately powered equivalence studies conducted in clinically relevant settings and samples. It would not be surprising if it emerges that different forms of psychopathology respond differently to the two forms of treatment delivery.

Not surprisingly, many important questions have yet to be answered. Here are some examples. First, as there have been few head-to-head comparisons of different digital interventions for the same mental health problem, it is not clear which ones are the most effective ones nor is their relative cost-effectiveness known (Donker et al., 2015). These are significant omissions since this information is needed by policy makers. Second, it is not known whether the functionality of a digital intervention has a bearing on its effectiveness. This too is an important omission as the answer has major implications for the design of future treatments. It is possible that the age of the user may be relevant with younger users complying better with more interactive treatments. The nature of the psychopathology being addressed may also need to be taken into account when designing interventions; for example, users with depression may struggle to complete interventions which require sustained concentration. In addition, there is a need for research on how these interventions work; who is accessing them; who benefits most; and whether the changes last. Also, more needs to be known about any negative effects of digital treatment (Rozental et al., 2014).

The evaluation of digital interventions poses intriguing challenges (Glasgow et al., 2014, Mohr et al., 2015). New interventions appear almost weekly, particularly app-based ones. Do all of them need to be evaluated or can one generalise from similar interventions? A related problem stems from the fact that digital interventions are easy to tweak. This raises the matter of at what point does a tweak result in what is essentially a “new intervention” that requires its own evaluation? The place of the conventional, somewhat ponderous, randomised controlled trial (RCT) is a particular concern in this fluid and fast-moving field. In the early stages of developing, testing and refining a digital intervention a variety of research designs may be of relevance (Collins et al., 2014, Collins et al., 2014, Glasgow et al., 2014, Lei et al., 2012), but once an intervention has been reasonably well defined RCTs are likely to be needed to establish its utility.

## 6 The use of digital treatments

It is far from clear how best to use digital treatments. To date they have been implemented in many different ways and in numerous different settings. Most have been accompanied by some form of support, but the nature of the support has varied greatly as have the qualifications and training of the person delivering it (who has been variously termed a “guide”, “coach”, “facilitator”, “therapist” or “clinician”). As a result, the research findings are difficult to integrate and interpret.

In Table 1 we propose a framework for classifying the ways that digital treatments may be used. It is based on whether the intervention is programme-led or clinician-led and, if there is external input, whether it is from a non-specialist practitioner or clinician.

### 6.1 Autonomous and supported digital treatment

The most scalable way of providing a digital treatment is without support (“autonomous digital treatment”) but, as noted earlier, the provision of support improves outcome. To obtain this benefit while minimising the loss of scalability that comes from providing support, the form that the support takes is of great importance. It needs to be from a non-specialist practitioner rather than a clinician. This can be achieved if the support solely involves enhancing the user's adherence to the digital intervention, a role that does not require extensive training or supervision. Thus in “supported digital treatment” (sometimes termed “guided self-help”^2^) the intervention remains programme-led with the digital programme delivering the intervention, not an outside expert.

There are many ways in which the support can be delivered. It can be via brief face-to-face sessions as exemplified by the use of supported self-help in the treatment of eating disorders (Wilson & Zandberg, 2012) or it can be via telephone or videoconferencing. Were large numbers of people to require support, a call centre model could be used as a single practitioner can support a range of different digital interventions at the same time since the role is essentially the same whatever the intervention. An alternative to spoken support is the provision of written messages sent via email or text messaging. This too is scalable but whether it is as effective as a spoken supportive dialogue is questionable.

### 6.2 Blended digital treatment

The concept of “blended treatment” is a new one. Generally, it refers to face-to-face treatments which include a digital intervention or component (Wentzel, van der Vaart, Bohlmeijer, & van Gemert-Pijnen, 2016) although the clinician involvement need not be literally face-to-face; for example, it could be via telephone or videconferencing. Blended treatment is gaining in popularity, a particularly early adopter being the Netherlands (Ruwaard & Kok, 2015).

Blended treatment can take a variety of forms. It can be programme-led as in what may be termed “supervised digital treatment”. This involves a digital programme delivering the intervention (as in autonomous digital treatment and supported digital treatment) while a clinician closely supervises its use and makes additional contributions as needed. These may include communicating regularly with the user (via whatever medium seems suitable), providing advice, controlling the pace at which the intervention is delivered, and deciding if and when further treatment modules should be made available. This role requires that the clinician be intimately familiar with the digital intervention and the treatment upon which it is based. Preliminary evidence suggests that supervised digital treatment may dramatically reduce the amount of clinician time needed without sacrificing effectiveness (Stott et al., 2013; Wild et al., 2016).

More commonly, blended treatment resembles conventional clinician-led treatment. In this case the digital intervention is used to make the treatment more efficient by taking over one or more tasks ordinarily undertaken by the clinician (for example, providing education) or it is used to make the treatment more effective as in the case of virtual reality-based exposure (Freeman et al., 2016, Valmaggia et al., 2016).

## 7 The use of digital treatments in community and clinical settings

Direct-to-user digital treatments have considerable potential as public health interventions (Fairburn and Patel, 2014, Munoz et al., 2016). Whether they are autonomous or accompanied by non-specialist support, they are scalable. If they are directly accessed via the internet, their potential reach is enormous and they may well circumvent many of the barriers to the receipt of help including stigma, shame, scarcity of local treatment resources and cost (Munoz et al., 2016).

A recent innovation is the online clinic, MindSpot being a leading example. It is a government-funded national online treatment service for Australian adults with anxiety or depression (Titov et al., 2015). It provides information and advice via email or over the telephone and, if indicated, it supplies digital treatments with or without external input. Online clinics like MindSpot are likely to proliferate. They have many advantages for users (for example, ease of access, and the convenience of having treatment sessions when and where it suits them) and for healthcare systems (for example, high patient throughput at low cost).

In standard clinical settings digital interventions may be used in a range of ways. Unguided and supported digital treatments are well suited for non-specialist settings such as primary care, although in practice uptake and use can be poor (Gilbody et al., 2015). In specialist settings blended treatment is likely to be the preferred option as the patients will be expecting input from a clinician.

A barrier to the use of blended treatment is the absence of guidance regarding when and how to implement it (Wentzel et al., 2016). This is important as blended treatment can take many forms, not all of which are advantageous (Kenter et al., 2015). For blended treatment to be rationally used and well implemented, it needs better operationalisation and further study.

## 8 The contribution of digital technology to the global dissemination of psychological treatments

The challenges to disseminating empirically supported psychological treatments are global; to our knowledge, their effective coverage does not exceed 50% in any country. Even this figure is likely to be a significant over-estimate. The coverage in low and middle income countries is much lower. A recent estimate from India and China, two relatively well resourced middle income countries, revealed a treatment gap exceeding 90% for common mental disorders and alcohol use disorders (Patel et al., 2016), the two mental health conditions for which psychological treatments are recommended as first line interventions by the mhGAP program (WHO, 2008).

Global mental health innovators have attempted to address two major barriers to reduce this gap, viz. their low acceptability due to contextual differences between the settings in which psychological treatments were developed and those in which they are to be used, and their low feasibility due to the lack of mental health professionals to deliver them (van Ginneken et al., 2013). This body of research has shown that psychological treatments are effective in widely different cultural and social contexts even when delivered by people with little or no prior mental health training (van Ginneken et al., 2013). However, there remain two significant barriers: the continuing reliance on face-to-face formats for training and supervision, and the low demand for mental health care in formal health care settings, not least due to the high levels of stigma attached to mental health problems. Digital technologies offer a genuine opportunity to leap-frog both barriers.

The Mental Health Care Innovation Network (www.mhinnovation.net), the largest repository of innovations in global mental health, has identified 38 innovations that are using digital technology to disseminate empirically supported interventions (24th April 2016). These include a wide range of approaches with distinct goals including the direct delivery of treatments such as cognitive behaviour therapy; social networking for mental health care provider supervision; digital platforms for service users to share experiences and support one another; case management systems for monitoring the delivery of care; telemedicine systems to provide mental health care to remote populations or supervision to front-line workers; screening and decision-support tools for front-line workers; video game interfaces to address psychopathology targets; and text messaging to motivate patients.

A systematic review of the evidence base on interventions to disseminate psychological treatments (Naslund et al., in preparation; protocol registration number: CRD42015027179) identified 44 studies that used digital technology for the treatment, diagnosis, or management of mental disorders, or for providing mental health training and education to health workers. The review found that these methods were well accepted and while it was concluded “that mobile and online technologies may hold significant potential to extend workforce capacity, reach forcibly displaced persons and refugees, support young people with mental disorders who are high users of mobile devices, and empower patients and families to take charge of their own care and to support each other through online communities” it was also noted that there has been little formal evaluation of their effectiveness. This is possibly the most important immediate priority for the field of global mental health, i.e., to identify best practices in each of the domains of technology-assisted delivery of psychological treatments.

There are a number of other challenges which will need to be addressed in the process of dissemination. Foremost amongst these are language (the vast majority of digital innovations are in English); the restricted coverage of internet-enabled devices; limited internet literacy in vast sections of the global population; and the lack of clearly defined regulatory procedures to ensure privacy and confidentiality of digital health data. Notwithstanding these limitations and challenges, we are bullish about the prospect of digital technology being transformative in improving the global availability of psychological treatments. Our enthusiasm is influenced by several factors: the ingenuity with which digital technology is being applied in diverse ways for diverse goals; the demonstrated successes of digital technology in a variety of other health care domains; the rapid growth in internet coverage and internet literacy, in particular among young people who are potentially the most important group for targeting psychological treatments for common mental health problems; and the ever-increasing speed of data access and the reducing cost of internet-enabled devices. .

While the “digital divide” undoubtedly remains a problem, particularly in low-resource settings, the divide is closing and there is no reason to think that this will not continue. Digital interventions that can be used without support are of particular importance as they have enormous potential to improve access, and additionally they have the value of being inherently empowering. They need to be optimised and “task sharing” needs to be expanded to embrace digital self-help. National and international organisations concerned with mental health need to endorse and support digital technologies as they are likely to be transformative. Above all, the international psychological treatment community must strive to engage digital entrepreneurs and innovators, particularly those who are championing initiatives in global health, to partner with them to exploit the many opportunities for using digital technology to transform mental health care worldwide.

## 9 Looking forward

Over the next decade or two much is likely to change. Digital interventions will gradually find their place within mental healthcare systems, and online clinics will become more commonplace. Digital assessment and treatment are likely to merge. Blended treatment may displace some conventional face-to-face treatment, and the limitations and negative effects of these innovations are likely to become evident. It is to be hoped that systems for evaluating, regulating and promoting these interventions will be developed thereby accelerating their appropriate use.

## Figures and Tables

**Table 1:** The use of digital treatments.

	Autonomous digital treatment	Supported digital treatment	Blended digital treatment	
Nature of the Intervention	Programme-led intervention	Programme-led intervention	Programme-led intervention	Clinician-led intervention
Characteristics	The use of a digital intervention with no external support	The use of a digital intervention with support from a non-specialist practitioner	The use of a digital intervention under the supervision of a clinician (“Supervised Digital Treatment”)	A clinician-led treatment which incorporates a digital intervention
External input	None	Support from a non-specialist practitioner	Supervision from a clinician	Treatment from a clinician
Setting	Community or clinical settings	Community or clinical settings	Clinical settings	Clinical settings

## Footnotes

1 Fifty years ago an equivalent sea-change began. The research methods of experimental psychology and clinical medicine began to be applied to the development and evaluation of psychological treatments. Among those who have contributed most to this revolution is G Terence (Terry) Wilson to whom this article is dedicated.

2 The first author introduced the notion of “guided self-help” in the 1990s. With hindsight the term is misleading as it implies that guidance is provided whereas the role of the “guide” in this scalable form of treatment is solely to support the user's adherence to the self-help intervention.

## References

[bib1] Abdullah S., Matthews M., Frank E., Doherty G., Gay G., Choudhury T. (2016). Automatic detection of social rhythms in bipolar disorder. Journal of the American Medical Informatics Association.

[bib2] Alfonsson S., Maathz P., Hursti T. (2014). Interformat reliability of digital psychiatric self-report questionnaires: A systematic review. Journal of Medical Internet Research.

[bib3] Andersson G. (2014). The internet and CBT: A clinical guide.

[bib4] Andersson G., Cuijpers P., Carlbring P., Riper H., Hedman E. (2014). Guided internet-based vs. face-to-face cognitive behavior therapy for psychiatric and somatic disorders: A systematic review and meta-analysis. World Psychiatry.

[bib5] Andersson G., Titov N. (2014). Advantages and limitations of Internet-based interventions for common mental disorders. World Psychiatry.

[bib6] van Ballegooijen W., Riper H., Cuijpers P., van Oppen P., Smit J.H. (2016). Validation of online psychometric instruments for common mental health disorders: A systematic review. BMC Psychiatry.

[bib7] Baumeister H., Reichler L., Munzinger M., Lin J. (2014). The impact of guidance on Internet-based mental health interventions — a systematic review. Internet Interventions.

[bib8] Ben-Zeev D., Schueller S.M., Begale M., Duffecy J., Kane J.M., Mohr D.C. (2015). Strategies for mHealth research: Lessons from 3 mobile intervention studies. Administration and Policy in Mental Health and Mental Health Services Research.

[bib9] Blackwell S.E., Browning M., Mathews A., Pictet A., Welch J., Davies J. (2015). Positive imagery-based cognitive bias modification as a web-based treatment tool for depressed adults: A randomized controlled trial. Clinical Psychological Science.

[bib10] Christensen H., Cuijpers P., Reynolds C.F. (2016). Changing the direction of suicide prevention research: A necessity for true population impact. JAMA Psychiatry.

[bib11] Christensen H., Griffiths K.M., Korten A. (2002). Web-based cognitive behavior therapy: Analysis of site usage and changes in depression and anxiety scores. Journal of Medical Internet Research.

[bib12] Collins L.M., Dziak J.J., Kugler K.C., Trail J.B. (2014). Factorial experiments: Efficient tools for evaluation of intervention components. American Journal of Preventive Medicine.

[bib13] Collins L.M., Nahum-Shani I., Almirall D. (2014). Optimization of behavioral dynamic treatment regimens based on the sequential, multiple assignment, randomized trial (SMART). Clinical Trials.

[bib14] Craske M.G., Rose R.D., Lang A., Welch S.S., Campbell-Sills L., Sullivan G. (2009). Computer-assisted delivery of cognitive behavioral therapy for anxiety disorders in primary-care settings. Depression and Anxiety.

[bib15] Cuijpers P., Donker T., van Straten A., Li G., Andersson G. (2010). Is guided self-help as effective as face-to-face psychotherapy for depression and anxiety disorders? A systematic review and meta-analysis of comparative outcome studies. Psychological Medicine.

[bib16] Donker T., Bennett K., Bennett A., Mackinnon A., van Straten A., Cuijpers P. (2013). Internet-delivered interpersonal psychotherapy versus internet-delivered cognitive behavioral therapy for adults with depressive symptoms: Randomized controlled noninferiority trial. Journal of Medical Internet Research.

[bib17] Donker T., Blankers M., Hedman E., Ljótsson B., Petrie K., Christensen H. (2015). Economic evaluations of internet interventions for mental health: A systematic review. Psychological Medicine.

[bib18] Donker T., Petrie K., Proudfoot J., Clarke J., Birch M.-R., Christensen H. (2013). Smartphones for smarter delivery of mental health programs: A systematic review. Journal of Medical Internet Research.

[bib19] Eichhorn S., Matkin G.W. (2016). Massive open online courses, big data, and education research. New Directions for Institutional Research.

[bib20] Fairburn C.G., Cooper Z. (2011). Therapist competence, therapy quality, and therapist training. Behaviour Research and Therapy.

[bib21] Fairburn C.G., Patel V. (2014). The global dissemination of psychological treatments: A road map for research and practice. American Journal of Psychiatry.

[bib22] Faurholt-Jepsen M., Vinberg M., Frost M., Christensen E.M., Bardram J., Kessing L.V. (2014). Daily electronic monitoring of subjective and objective measures of illness activity in bipolar disorder using smartphones– the MONARCA II trial protocol: A randomized controlled single-blind parallel-group trial. BMC psychiatry.

[bib23] Freeman D. (2008). Studying and treating schizophrenia using virtual reality: A new paradigm. Schizophrenia Bulletin.

[bib24] Freeman D., Bradley J., Antley A., Bourke E., DeWeever N., Evans N. (2016). Virtual reality in the treatment of persecutory delusions: Randomised controlled experimental study testing how to reduce delusional conviction. British Journal of Psychiatry.

[bib25] Gilbody S., Littlewood E., Hewitt C., Brierley G., Tharmanathan P., Araya R. (2015). Computerised cognitive behaviour therapy (cCBT) as treatment for depression in primary care (REEACT trial): Large scale pragmatic randomised controlled trial. British Medical Journal.

[bib26] van Ginneken N., Tharyan P., Lewin S., Rao G.N., Meera S., Pian J. (2013). Non-specialist health worker interventions for the care of mental, neurological and substance-abuse disorders in low- and middle-income countries. Cochrane Database of Systematic Reviews.

[bib27] Glasgow R.E., Phillips S.M., Sanchez M.A. (2014). Implementation science approaches for integrating eHealth research into practice and policy. International Journal of Medical Informatics.

[bib28] Hart L.M., Granillo M.T., Jorm A.F., Paxton S.J. (2011). Unmet need for treatment in the eating disorders: A systematic review of eating disorder specific treatment seeking among community cases. Clinical Psychology Review.

[bib29] James E.L., Bonsall M.B., Hoppitt L., Tunbridge E.M., Geddes J.R., Milton A.L. (2015). Computer game play reduces intrusive memories of experimental trauma via reconsolidation-update mechanisms. Psychological Science.

[bib30] Kenter R.M.F., van de Ven P.M., Cuijpers P., Koole G., Niamat S., Gerrits R.S. (2015). Costs and effects of internet cognitive behavioral treatment blended with face-to-face treatment: Results from a naturalistic study. Internet Interventions.

[bib31] Kleiboer A., Donker T., Seekles W., van Straten A., Riper H., Cuijpers P. (2015). A randomized controlled trial on the role of support in Internet-based problem solving therapy for depression and anxiety. Behaviour Research and Therapy.

[bib32] Lei H., Nahum-Shani I., Lynch K., Oslin D., Murphy S.A. (2012). A “SMART” design for building individualized treatment sequences. Annual Review of Clinical Psychology.

[bib33] Ly K.H., Trüschel A., Jarl L., Magnusson S., Windahl T., Johansson R. (2014). Behavioural activation versus mindfulness-based guided self-help treatment administered through a smartphone application: A randomised controlled trial. BMJ Open.

[bib34] McHugh R.K., Barlow D.H., McHugh R.K., Barlow D.H. (2012). Training in evidence-based psychological interventions. Dissemination and implementation of evidence-based psychological interventions.

[bib35] Merry S.N., Stasiak K., Shepherd M., Frampton C., Fleming T., Lucassen M.F.G. (2012). The effectiveness of SPARX, a computerised self help intervention for adolescents seeking help for depression: Randomised controlled non-inferiority trial. British Medical Journal.

[bib36] Mohr D.C., Cuijpers P., Lehman K. (2011). Supportive accountability: A model for providing human support to enhance adherence to eHealth interventions. Journal of Medical Internet Research.

[bib37] Mohr D.C., Schueller S.M., Riley W.T., Brown C.H., Cuijpers P., Duan N. (2015). Trials of intervention principles: Evaluation methods for evolving behavioral intervention technologies. Journal of Medical Internet Research.

[bib38] Munoz R.F., Bunge E.L., Chen K., Schueller S.M., Bravin J.I., Shaughnessy E.A. (2016). Massive open online interventions: A novel model for delivering behavioral-health services worldwide. Clinical Psychological Science.

[bib39] Murnane E.L., Cosley D., Chang P., Guha S., Frank E., Gay G. (2016). Self-monitoring practices, attitudes, and needs of individuals with bipolar disorder: Implications for the design of technologies to manage mental health. Journal of the American Medical Informatics Association.

[bib40] Patel V., Xiao S., Chen H., Hanna F., Jotheeswaran A.T., Luo D. (2016). The magnitude of and health system responses to the mental health treatment gap in adults in India and China. Lancet.

[bib41] Pots W.T.M., Fledderus M., Meulenbeek P.A.M., ten Klooster P.M., Schreurs K.M.G., Bohlmeijer E.T. (2016). Acceptance and commitment therapy as a web-based intervention for depressive symptoms: Randomised controlled trial. British Journal of Psychiatry.

[bib42] Richards D., Richardson T. (2012). Computer-based psychological treatments for depression: A systematic review and meta-analysis. Clinical Psychology Review.

[bib43] Riek L.D. (2016). Robotics technology in mental health care. Artificial intelligence in behavioral and mental health care.

[bib44] Rozental A., Andersson G., Boettcher J., Ebert D.D., Cuijpers P., Knaevelsrud C. (2014). Consensus statement on defining and measuring negative effects of Internet interventions. Internet interventions.

[bib45] Ruwaard J., Kok R.N. (2015). Wild west eHealth: Time to hold our horses?. European Health Psychologist.

[bib46] Saeb S., Zhang M., Karr C.J., Schueller S.M., Corden M.E., Kording K.P. (2015). Mobile phone sensor correlates of depressive symptom severity in daily-life behavior: An exploratory study. Journal of Medical Internet Research.

[bib47] Spijkerman M.P.J., Pots W.T.M., Bohlmeijer E.T. (2016). Effectiveness of online mindfulness-based interventions in improving mental health: A review and meta-analysis of randomised controlled trials. Clinical Psychology Review.

[bib48] Stott R., Wild J., Grey N., Liness S., Warnock-Parkes E., Commins S. (2013). Internet-delivered cognitive therapy for social anxiety disorder: A development pilot series. Behavioural and Cognitive Psychotherapy.

[bib49] Titov N., Dear B.F., Schwencke G., Andrews G., Johnston L., Craske M.G. (2011). Transdiagnostic internet treatment for anxiety and depression: A randomised controlled trial. Behaviour Research and Therapy.

[bib50] Titov N., Dear B.F., Staples L.G., Bennett-Levy J., Klein B., Rapee R.M. (2015). MindSpot clinic: An accessible, efficient, and effective online treatment service for anxiety and depression. Psychiatric Services.

[bib51] Valmaggia L.R., Latif L., Kempton M.J., Rus-Calafell M. (2016). Virtual reality in the psychological treatment for mental health problems: An systematic review of recent evidence. Psychiatry Research.

[bib52] Van Spijker B.A.J., Van Straten A., Kerkhof A.J.F.M. (2014). Effectiveness of online self-help for suicidal thoughts: Results of a randomised controlled trial. PLoS One.

[bib54] Wentzel J., van der Vaart R., Bohlmeijer E.T., van Gemert-Pijnen J.E.W.C. (2016). Mixing online and face-to-face therapy: How to benefit from blended care in mental health care. JMIR Mental Health.

[bib56] Wild J., Warnock-Parkes E., Grey N., Stott R., Wiedemann M., Canvin L. (2016). Internet-delivered cognitive therapy for PTSD: A development pilot series. European Journal of Psychotraumatology.

[bib55] Wilson G.T., Zandberg L.J. (2012). Cognitive–behavioral guided self-help for eating disorders: Effectiveness and scalability. Clinical Psychology Review.

[bib57] World Health Organization (2008). Mental Health Gap Action Programme (mhGAP): Scaling up care for mental, neurological and substance abuse disorders.

